# Tensile Failure Behaviors of Adhesively Bonded Structure Based on In Situ X-ray CT and FEA

**DOI:** 10.3390/ma16247609

**Published:** 2023-12-12

**Authors:** Jiawen Tang, Bo Niu, Yu Cao, Yayun Zhang, Donghui Long

**Affiliations:** 1 State Key Laboratory of Chemical Engineering, East China University of Science and Technology, Shanghai 200237, China; 15026846480@163.com (J.T.); 18964036521@163.com (Y.C.); yy.zhang@ecust.edu.cn (Y.Z.); 2 Key Laboratory for Specially Functional Materials and Related Technology, East China University of Science and Technology, Shanghai 200237, China

**Keywords:** adhesively bonded structure, tensile failure, CT analysis, finite element analysis (FEA)

## Abstract

Adhesive bonding plays a pivotal role in structural connections, yet the bonding strength is notably affected by the presence of pore defects. However, the invisibility of interior pores severely poses a challenge to understanding their influence on tensile failure behaviors under loading. In this study, we present a pioneering investigation into the real-time micro-failure mechanisms of adhesively bonded structures using in situ X-ray micro-CT. Moreover, the high-precision finite element analysis (FEA) of stress distribution is realized by establishing the real adhesive layer model based on micro-CT slices. The findings unveil that pores induce stress concentration within the adhesive layer during the tensile process, with stress levels significantly contingent upon pore sizes rather than their specific shapes. Consequently, larger pores initiate and propagate cracks along their paths, ultimately culminating in the failure of adhesively bonded structures. These outcomes serve as a significant stride in elucidating how pore defects affect the bonding performance of adhesively bonded structures, offering invaluable insights into their mechanisms.

## 1. Introduction

Adhesive bonding is a structural connection technology utilizing the mechanical bonding force, physical adsorption force, and chemical bonding force generated by the adhesive on joint surfaces [1,2,3]. Compared with traditional rivet bonding and welding technology, adhesive bonding exhibits the advantages of minor deformation, uniform stress distribution, and simple processing, and, thus, it has been widely used in industrial fields [4,5]. However, due to volatiles, entrained gases (principally air and water vapor), or insufficient adhesive being applied, the adhesive layer inevitably contains bonding defects such as pores and bubbles after curing [6]. The evolution of defect structures under loading will reduce the effective bonding area and cause stress concentration, thereby resulting in the failure of adhesively bonded structures [7]. Therefore, deeply revealing the evolution behaviors of defect structures under loading is of great significance for assessing the bonding performance of adhesively bonded structures.

Significant efforts have been made for studying how defect structures affect the bonding performance of adhesively bonded structures; the variable-based test methods are the most extensively employed due to their ease of use. For instance, researchers artificially introduced defect structures into the adhesive layer and examine their impact on bonding strength by manipulating defect properties (such as defect volume, defect location, defect form, etc.) [8,9,10]. However, it is difficult to reveal the microscopic influence mechanisms using such experimental approaches based on macromechanical testing and fractured morphology observation. To address this issue, numerous simulation studies, such as finite element analysis (FEA), have also been carried out. For instance, based on FEA, it is possible to clearly illustrate how defect location, number, and form affect the stress distribution of the adhesive layer during the shear process [11]. But this modeling method cannot reproduce the real structure of the adhesive layer. In addition, existing researches, both based on experiment and simulation methods, are generally confined to artificially introduced defects. And there are few studies focused on the naturally formed defects during the bonding process, limited by the monitoring and visualization of interior defects inside the adhesively bonded structures [12].

Recently, X-ray micro-computed tomography (micro-CT), which can precisely provide three-dimensional (3D) microstructure inside materials, has been widely adopted to reconstruct multiphase materials, such as porous materials [13], particle-reinforced composites [14], interior pore characteristics in the epoxy resin adhesive layer [15,16], etc. The in situ X-ray micro-CT under loading, which conducts X-ray scanning during the loading process, has been developed over time into a powerful instrument for investigating the failure behaviors of materials in real time; for example, the microstructure evolution of short fiber-reinforced composites [17] and damage behaviors of 3D braided composites [18,19,20]. Therefore, by monitoring the real-time evolution behaviors of defects (through experiments) and reconstructing the 3D FEA model (through simulation), the in situ X-ray micro-CT technology shows significant prospects for revealing the underlying micro-failure mechanisms of adhesively bonded structures. Different from the above test methods and FEA methods, in situ X-ray micro-CT combined with FEA can not only monitor the entire macroscopic fracture process in real time and reveal the microscopic fracture mechanism, but can also realize the real structure of adhesive layer structure modeling through the subsequent analysis of CT data, providing a real adhesive layer model for FEA. Additionally, this method can be applied not only to the study of pore defects, but also to the study of the interface between adhesive and adherend. In addition, by improving the accuracy of CT scanning, the internal microstructure of the adhesive can be accurately analyzed, and the reinforcing effect of the filler type and the particle size and shape on the fracture process in the adhesive layer can be analyzed. Therefore, this method has a high application prospect.

In this work, the real-time micro-failure mechanisms of adhesively bonded structures are comprehensively investigated based on in situ X-ray micro-CT and FEA to thoroughly reveal the evolution behaviors of pore defects under loading. Based on in situ X-ray micro-CT, the structural evolution of pores during the tensile shear process and its impact on crack propagation are tracked and monitored. Furthermore, the combination of FEA and the 3D model reconstructed by X-ray micro-CT slices is used to investigate how pore defects affect the stress distribution in the shear tensile process. The results reveal that pores lead to the stress concentration in the adhesive layer during tensile process, and the stress is substantially influenced by pore size and less so by form. Therefore, larger pores will cause the crack’s initiation and propagation along the pore path, thus leading to the failure of adhesively bonded structures.

## 2. Experimental Method

### 2.1. In Situ X-ray Micro-CT under Tensile

The target adhesive was KH-RTV-400 silicone adhesive (equipped with a specific curing agent), created by the Institute of Chemistry, Chinese Academy of Sciences (Beijing China), and the adhesive diluent was cyclohexane. The adhesive was widely used in the field of thermal protection, mainly for the adhesion of thermal protection materials to aircraft surfaces because of its excellent heat resistance. The adhesive mixture was made using the mass ratio of 100:4:10 for adhesive, curing agent, and diluent, which was given by the Institute of Chemistry, Chinese Academy of Sciences, then the mixture was used to bond the aluminum alloy sheet, which has dimensions of 10 × 5 × 2 mm^3^ (length, breadth, and height). To observe pore defects more clearly, the adhesive was applied to the 5 × 5 mm^2^ overlap region of the bonding joint and the thickness of the adhesive layer was controlled at 0.8 mm. The 3D structure evolution of the adhesive layer was tracked and detected using an industrial micro-CT (Zeiss, Xradia520 Versa, Oberkochen, Germany). A micromechanical test apparatus (CT5000, Deben, Woolpit, UK, 5 kN) with X-ray sources and a detector on either side was used to scan the sample with a loading rate of 0.1 mm/min and a voxel resolution of 2.0 µm.

### 2.2. Chromatographic Image Processing and 3D Reconstruction

**Figure 1:**
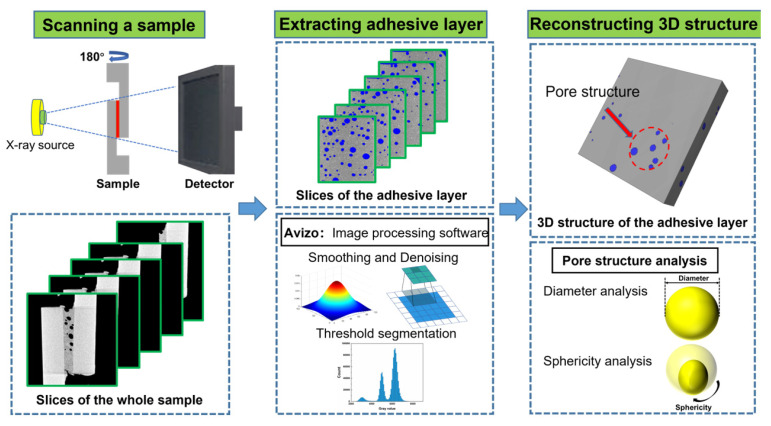
Reconstruction process of 3D pore structure based on X-ray micro-CT.

As seen in Figure 1, the adhesive layer was extracted from the collected original 2D CT slices, and Avizo 2021 software was used to process the 2D CT slices for noise reduction to smooth out and suppress noise interference during the shooting process and improve picture contrast. The 3D structure of the adhesive layer was reconstructed using binarization threshold segmentation technology, and the properties of the pore structure were examined.

### 2.3. Finite Element Modeling

To establish an FEA model, the extracted adhesive layer structure (actual size: 4.62 × 4.71 × 0.87 mm^3^) was imported into Abaqus 2021 software. The elastic modulus and Poisson’s ratio of adhesive were 6 MPa and 0.47 [21], respectively. The model was meshed using large-scale hexahedral voxel mesh with the number of 2,625,000, and the size of each mesh is 36.9 µm × 22.4 µm × 8.7 µm. The sample was subjected to shear action during the whole experiment, and considering that no interfacial debonding occurred, it could be considered that the adhesive layer was subjected to shear action on the upper and lower surfaces. Therefore, the Dirichlet boundary conditions of the model were as follows: one end was fixed, while the other end was subjected to a displacement of 2 mm along the y-axis, which simulated shear action during the experiment. This displacement load was determined according to the maximum displacement at the time of tensile failure.

## 3. Results and Discussion

### 3.1. Micro-Failure Mechanisms Based on In Situ X-ray Micro-CT

**Figure 2:**
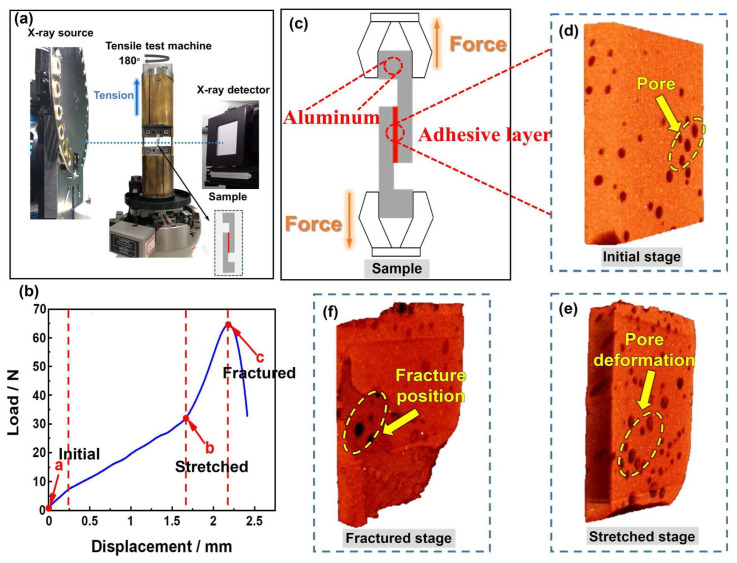
(**a**) Diagram of in situ X-ray CT setup and test; (**b**) displacement–load curve of tensile pre-test; (**c**) shape of the in situ tensile specimen; and (**d**–**f**) the original 3D structure of adhesive layers at different stages.

During the entire tensile process, the micro-failure mechanisms of adhesively bonded structures are further revealed by using X-ray micro-CT to trace the evolution of pore structure. The displacement–load curve of the in situ test (Figure 2b) reveals that the curve appears in three stages. This is because the modulus of rubber materials will manifest in three stages during the stretching process: the first stage is modulus decline, the second stage is constant modulus, and the third stage is modulus increase [22]. The Payne effect [23], which refers to the dynamic modulus of rubber material decreasing with increasing strain, can explain the drop in modulus with modest displacement. The primary cause of the drop in modulus is the internal filler network structure of the rubber material, which is harmed during the tensile process. Rubber hardening under heavy strain is the cause of the rising modulus under considerable displacement [24].

Based on the displacement–load curve, three stages—the initial stage, stretched stage, and the fractured stage—are chosen for CT scanning. The acquired 2D CT slices are subjected to noise reduction and threshold segmentation using the commercial software Avizo 2019.2. The 3D reconstruction of the adhesive layer structure is also finished using it.

**Figure 3:**
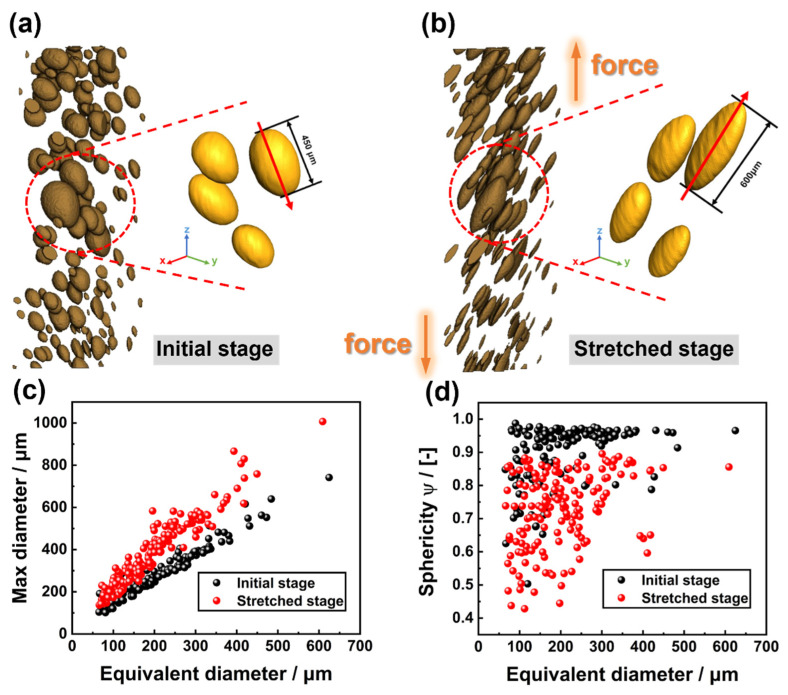
(**a**,**b**) Pore structure of the initial stage and stretched stage; (**c**) distribution of maximum pore diameter; and (**d**) distribution of pore sphericity.

The pore structure evolution in the adhesive layer at the initial stage and stretched stage is analyzed (Figure 3), which is highlighted by the distribution of maximum diameter and sphericity *Ψ* [25] (Equation (1)).

(1) $$\Psi =\frac{\pi^{\frac{1}{3}}{\left(6V_{P}\right)}^{\frac{2}{3}}}{A_{P}},$$

where $V_{P}$ is the volume of the pores and $A_{P}$ is the area of the pores.

At the initial stage, the majority of pores are spherical and have sphericity values that are close to 1. While at the stretched stage, the sphericity drastically reduces and the pores take on a flat ellipsoid shape. The average maximum diameter increases from 287 µm to 388 µm at the same time as the diameter of the pore increases with pore deformation. In the extracted pore structure (Figure 3a,b), this phenomenon could be seen more clearly. The maximum diameter of a pore increases from 450 µm to 600 µm when it changes from spherical in the initial stage to flat ellipsoid in the stretched stage.

**Figure 4:**
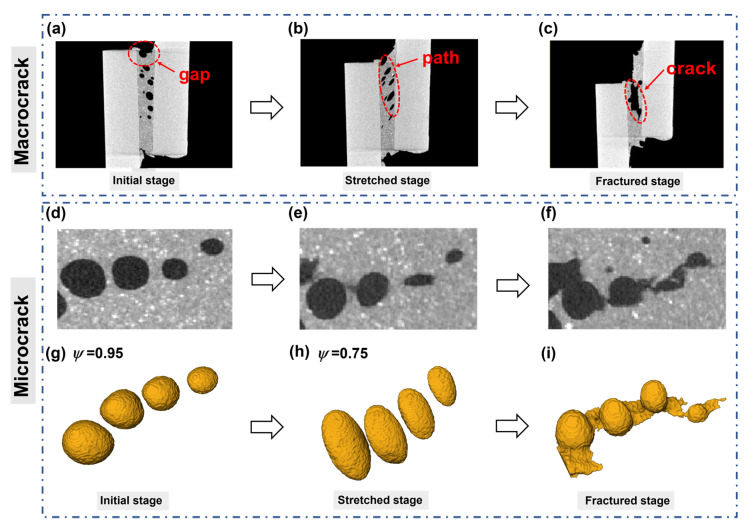
(**a**–**c**) 2D CT slices of macrocrack evolution; (**d**–**f**) 2D CT slices of microcrack evolution; and (**g**–**i**) 3D reconstruction of microcrack evolution.

The 2D CT slices at various stages are displayed in Figure 4 to show the crack propagation in the bonded structure under in situ tensile shear. It demonstrates that the pores at one end of the adhesive layer are connected to the beginning of the macrocrack (Figure 4a). Additionally, the presence of pores causes a sphere-shaped gap to emerge at the edge of the adhesive layer. This gap turns into the first point of the crack during the tensile shear process, and the crack gradually spreads along interior pores, thus resulting in the failure of the bonded structure.

Microcracks created by four pores are extracted in Figure 4d–f to more clearly illustrate how pores affect crack initiation and propagation. The four pores exhibit various morphologies at various stages, as seen in the 2D slices. At the initial stage, the pores are round without a shear effect. The pores then extend as a result of the shear pressure and take on a squashed shape. At the fractured stage, pores finally unite to form microcracks. The 3D reconstructed morphologies (Figure 4g–i) make it easier to see how the crack evolved. Under shear strain, the round pores (initial stage) deform into an ellipsoidal structure (stretched stage). As the tensile load increases, pores begin to break and eventually form a microcrack at the fractured stage.

**Figure 5:**
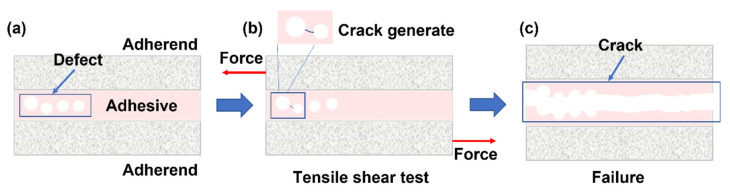
Schematic illustration of the fracture mechanism of the joint with pore defects. (**a**–**c**) different stages of tensile shear test.

The occurrence of adhesive failure around pore defects could be explained by Figure 5. In the process of tensile shear test, the stress concentration near the pores occurs easily in the area with pore defects. However, in areas far from the defect, the uneven pressure distribution is alleviated. Therefore, due to the existence of pores, cracks are easily generated along the interface around the pores, and once the tip cracks are generated, because the surrounding pores will also produce stress concentration, the tip cracks will continue to expand inward along the stress concentration area. The cracks will then spread to the bonding layer, far away from the pore defects, because the interface strength exceeds the cohesion strength of the bonding layer, far away from the defect area.

### 3.2. Finite Element Analysis

**Figure 6:**
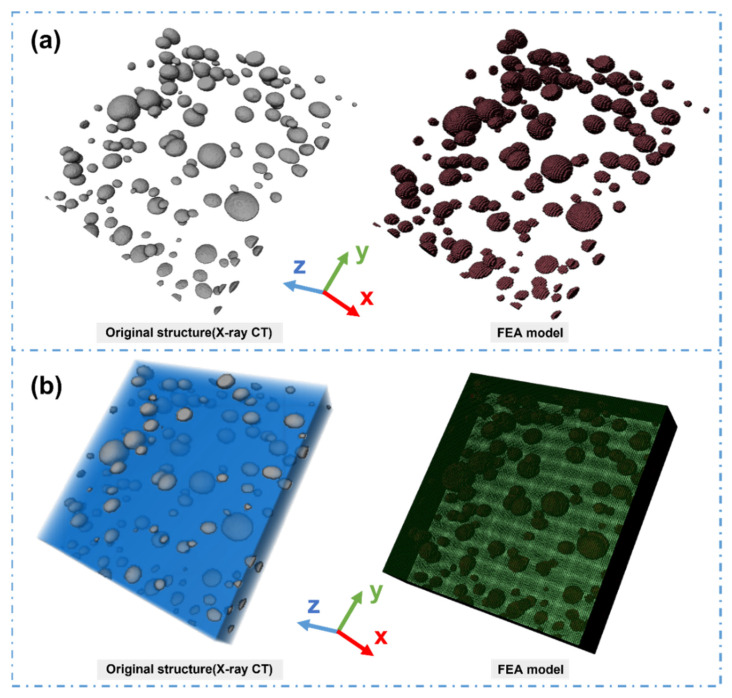
High-precision FEA model of adhesive layer constructed based on X-ray micro-CT: (**a**) extraction and meshing of the pore structure in the adhesive layer; and (**b**) construction and meshing of the high-precision model of the adhesive layer.

According to the aforementioned findings, the pore structure is closely related to the bonding failure inside the adhesive layer. The initial adhesive layer of the initial stage is chosen to illustratively demonstrate the impact of pore structure on the stress–strain distribution of the adhesive layer during tensile shear operation. To establish a high-precision model, threshold segmentation, noise reduction, and other treatments are carried out. Abaqus 2021 software is then used to establish the FEA model, meshing, boundary setting, and load loading. The results are displayed in Figure 6. The original X-ray micro-CT model of the adhesive layer and the established FEA model closely match each other. Therefore, this series of treatments can better retain the real pore morphology in the adhesive layer.

**Figure 7:**
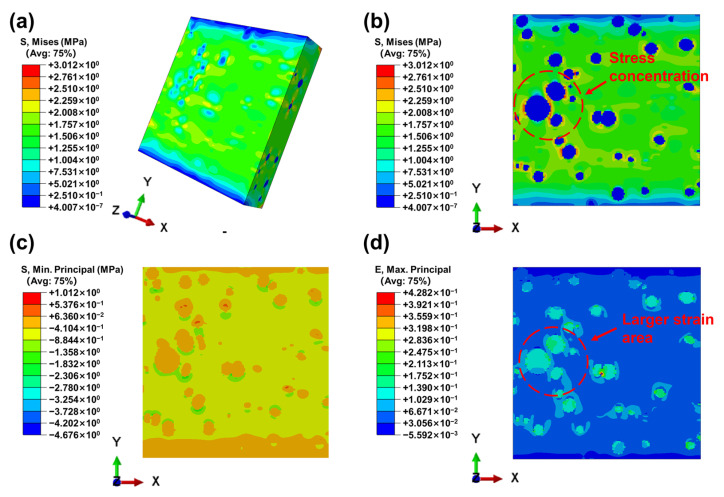
Finite element analysis results of the adhesive layer: (**a**) stress contour of the whole model; (**b**) stress contour near the pores; (**c**) minimum principal stress contour near the pores; and (**d**) strain contour near the pores.

Figure 7 displays the stress and strain contours of the entire model as well as those close to the pores under a tensile displacement of 2 mm. It is clear that, except for the two ends of the model, the stress distribution throughout the entire adhesive layer is rather homogeneous. In the adhesive layer, stress concentration also happens close to the pores and the equator of the pores experiences the greatest stress. Meanwhile, the sites of stress concentration are connected by the presence of nearby pores. The strain in the adhesive layer is also mainly concentrated near the pores [26]. According to the simulation results, cracks in the adhesive layer will start to appear close to the pores. The nearby pores will make it simpler for the damage to connect, which will lead to crack propagation along the pores. This analysis is in good agreement with experimental phenomena in X-ray micro-CT.

**Figure 8:**
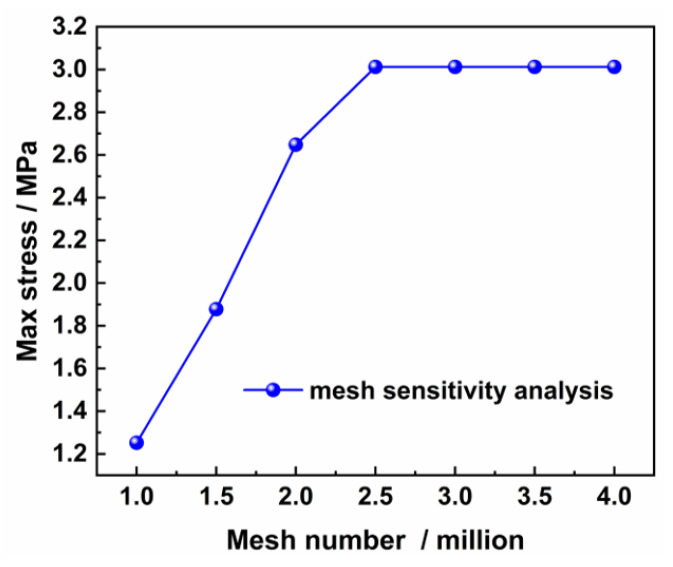
Mesh sensitivity analysis.

In order to exclude the influence of mesh size and number on FEA analysis, we perform a mesh sensitivity analysis, as shown in Figure 8. The maximum stress value of the model is selected as the target characteristic value, and the effect of the number of mesh on the maximum stress value is studied. The results show that the maximum stress value increases with the increase in the number of mesh. As the number of mesh increases to more than 2.5 million, the maximum stress value does not change, indicating that the mesh sensitivity has been excluded.

**Figure 9:**
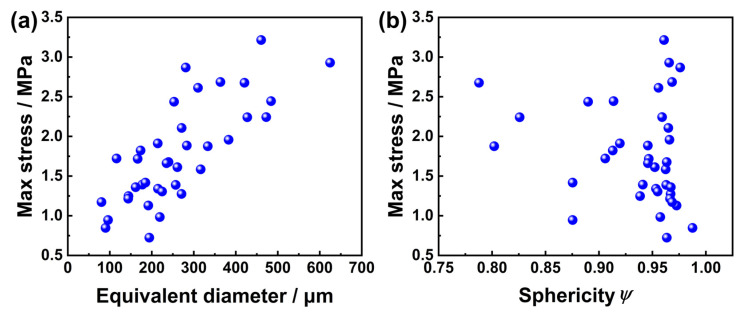
Relationship between maximum stress value and characteristic parameter of pores: (**a**) equivalent diameter; and (**b**) sphericity.

The pores in the middle of the adhesive layer are chosen to remove the impact of pore placement on the maximum stress value. How the equivalent diameter and sphericity of the pores and the maximum stress value near the pores relate to each other is examined. Sphericity does not appear to have a clear relationship with the maximum stress value, as evidenced by the results, as shown in Figure 9. Although, in the existing literature, some scholars have pointed out that the shape of the defect can also affect the magnitude of the stress value [27,28,29,30], this phenomenon is generally studied by artificially introducing defects of different shapes—circle, square and triangle. The shape difference of defects is huge, which leads to a large difference in stress values. However, in this paper, the defects are naturally formed, and the shapes are mostly spherical, with little difference in sphericity, so the stress value has no obvious relationship. Additionally, when the pore diameter rises, the maximum stress value increases noticeably close to the pores. This phenomenon arises because as the defect size increases, more load has to be transferred by the remaining adhesive layer near the pores [31]. Additionally, Crocombe posited the adhesive’s global yield as a principal mechanism governing the failure of adhesive joints [32]. Failure will happen when all the parts of the adhesive layer reach a state in which they can no longer sustain a significant increase in applied load. Thus, it can be predicted that the larger the pores, the higher the stress value of the adhesive layer near the pores. And once the stress value there exceeds the maximum value that can be borne, the damage will appear. The outcome provides a clear explanation of how the aforementioned microcracks form: damage first manifests itself close to the large pores, then slowly spreads to the neighboring pores until finally forming the microcracks.

## 4. Conclusions

In this paper, for the first time, the in situ X-ray micro-CT combined with FEA was used to reveal the microscopic failure mechanism of bonded structures, and the following conclusions can be drawn:

- In situ X-ray micro-CT tensile test monitored the comprehensive process of tensile failure in adhesively bonded structures in real time. Based on three-dimensional reconstruction technology, the microstructures of the adhesive layer across different stages of the tensile process—initial, stretching, and fracture phases—were visible.
- In the initial phase, naturally formed pores within the adhesive layer predominantly exhibited an ellipsoidal morphology. As load was applied, these pore structures transformed from an ellipsoid to a flat ellipsoid in the same direction as the stress, and the sphericity significantly diminished.
- Macrocracks started at the gap created by the pores and propagated along the pores’ path. Microcracks developed from crack connections between the pores. This phenomenon arose due to stress concentration in areas with pore defects, leading to crack initiation near these pores. Once the tip crack was generated, it would continue to expand inward along the stress concentration area, and then spread to the adhesive layer far away from the pore defects, eventually leading to the tensile failure.
- The results of the FEA revealed stress concentrations near the pores, progressively linking during the tensile process, thereby initiating cracks. The shape of the pores minimally impacted stress, whereas the size of the pores significantly influenced stress distribution, because as the defect size increased, more load was transferred by the remaining adhesive layer near the pores.

## Data Availability

Date are available on request.

## References

[1] Baldan A. (2012). Adhesion phenomena in bonded joints. Int. J. Adhes. Adhes..

[2] Komvopoulos K. (2003). Adhesion and friction forces in microelectromechanical systems: Mechanisms, measurement, surface modification techniques, and adhesion theory. J. Adhes. Sci. Technol..

[3] Lo C.T., Laabs F.C., Narasimhan B. (2004). Interfacial adhesion mechanisms in incompatible semicrystalline polymer systems. J. Polym. Sci. Pol. Phys..

[4] Shang X., Marques E.A.S., Machado J.J.M., Carbas R.J.C., Jiang D., Da Silva L.F.M. (2019). Review on techniques to improve the strength of adhesive joints with composite adherends. Compos. Part B Eng..

[5] Ouyang X., Chen C. (2023). Research on the joining of aluminum alloy and high-strength steel by dieless clinched-adhesive processes. J. Mater. Res. Technol..

[6] Adams R. (1988). A review of defect types and nondestructive testing techniques for composites and bonded joints. NDT&E Int..

[7] Chew H.B., Guo T.F., Cheng L. (2008). Influence of Nonuniform Initial Porosity Distribution on Adhesive Failure in Electronic Packages. IEEE T Compon. Pack T.

[8] Zhang T., Meng J., Pan Q., Sun B. (2019). The influence of adhesive porosity on composite joints. Compos. Commun..

[9] Fame C.M., Ramôa Correia J., Ghafoori E., Wu C. (2021). Damage tolerance of adhesively bonded pultruded GFRP double-strap joints. Compos. Struct..

[10] Ghasemvand M., Behjat B., Ebrahimi S. (2023). Experimental investigation of the effects of adhesive defects on the strength and creep behavior of single-lap adhesive joints at various temperatures. J. Adhes..

[11] Elhannani M., Madani K., Legrand E., Touzain S., Feaugas X. (2017). Numerical analysis of the effect of the presence, number and shape of bonding defect on the shear stresses distribution in an adhesive layer for the single-lap bonded joint; Part 1. Aerosp. Sci. Technol..

[12] Carrere N., Doitrand A., Martin E., Leguillon D. (2021). Theoretical study based on 2D assumptions of the influence of small pores on crack initiation in adhesively bonded joints. Int. J. Adhes. Adhes..

[13] Maire E. (2012). X-ray Tomography Applied to the Characterization of Highly Porous Materials. Annu. Rev. Mater. Res..

[14] Gad S.I., Attia M.A., Hassan M.A., El Shafei A.G. (2021). A random microstructure-based model to study the effect of the shape of reinforcement particles on the damage of elastoplastic particulate metal matrix composites. Ceram. Int..

[15] Du Plessis A., Yadroitsava I., Yadroitsev I. (2020). Effects of defects on mechanical properties in metal additive manufacturing: A review focusing on X-ray tomography insights. Mater. Des..

[16] Dumont V., Badulescu C., Stamoulis G., Adrien J., Maire E., Lefèvre A. (2020). On the influence of mechanical loadings on the porosities of structural epoxy adhesives joints by means of in-situ X-ray microtomography. Int. J. Adhes. Adhes..

[17] Hu X., Fang J., Xu F., Dong B., Xiao Y., Wang L. (2016). Real internal microstructure based key mechanism analysis on the micro-damage process of short fibre-reinforced composites. Sci. Rep..

[18] Ge L., Li H., Zhong J., Zhang C., Fang D. (2021). Micro-CT based trans-scale damage analysis of 3D braided composites with pore defects. Compos. Sci. Technol..

[19] Zhang D., Liu Y., Liu H., Feng Y., Guo H., Hong Z. (2021). Characterisation of damage evolution in plain weave SiC/SiC composites using in situ X-ray micro-computed tomography. Compos. Struct..

[20] Niu B., Shen H., Li T., Zhang H., Qian Z., Cao Y. (2022). 2.5D quartz fabric reinforced nanoporous phenolic composites with weakened heat transfer and optimized mechanical properties. Compos. Sci. Technol..

[21] Tong W.M., Bai S.J., Xing H., Nie Y.S. (2021). Research on the Application of RTV566 Adhesive in Space Infrared Cryogenic Lens. Infrared.

[22] Stricher A.M., Rinaldi R.G., Barrès C., Ganachaud F., Chazeau L. (2015). How I met your elastomers: From network topology to mechanical behaviours of conventional silicone materials. RSC Adv..

[23] Li X., Tian C., Li H., Liu X., Zhang L., Hong S. (2022). Combined effect of volume fractions of nanofillers and filler-polymer interactions on 3D multiscale dispersion of nanofiller and Payne effect. Compos. Part A Appl. Sci. Manuf..

[24] Mooney M. (1940). A Theory of Large Elastic Deformation. J. Appl. Phys..

[25] Wadell H. (1935). Volume, Shape, and Roundness of Quartz Particles. J. Geol..

[26] Yang H., Xiao R., Yang Z., Lei D. (2019). Experimental characterization and finite element modeling the deformation behavior of rubbers with geometry defects. Polym. Test..

[27] Sam-Daliri O., Faller L.-M., Farahani M., Roshanghias A., Araee A., Baniassadi M., Oberlercher H., Zangl H. (2019). Impedance analysis for condition monitoring of single lap CNT-epoxy adhesive joint. Int. J. Adhes. Adhes..

[28] Sam-Daliri O., Faller L.-M., Farahani M., Zangl H. (2021). Structural health monitoring of adhesive joints under pure mode I loading using the electrical impedance measurement. Eng. Fract. Mech..

[29] Ghabezi P., Farahani M. (2017). Composite Adhesive-Bonded Joint Reinforcement by Incorporation of Nano-Alumina Particles. J. Appl. Comput. Mech..

[30] Adhikari K., Baral S., Kumar S., Behera R.K. (2023). Experimental study of the influence of pre-embedded defect shape and size on the performance of adhesively bonded single lap joints using Al-6061 adherends. Mater. Today Proc..

[31] Karachalios E.F., Adams R.D., Da Silva Lucas F.M. (2013). Strength of single lap joints with artificial defects. Int. J. Adhes. Adhes..

[32] Crocombe A.D. (1989). Global yielding as a failure criterion for bonded joints. Int. J. Adhes. Adhes..

